# In Vitro Cellular and Molecular Interplay between Human Foreskin-Derived Mesenchymal Stromal/Stem Cells and the Th17 Cell Pathway

**DOI:** 10.3390/pharmaceutics13101736

**Published:** 2021-10-19

**Authors:** Mehdi Najar, Makram Merimi, Wissam H. Faour, Catherine A. Lombard, Douâa Moussa Agha, Yassine Ouhaddi, Etienne M. Sokal, Laurence Lagneaux, Hassan Fahmi

**Affiliations:** 1 Laboratory of Clinical Cell Therapy, Jules Bordet Institute, Université Libre de Bruxelles, 1070 Brussels, Belgium; mnajar@ulb.ac.be (M.N.); Laurence.Lagneaux@ulb.be (L.L.); 2 Osteoarthritis Research Unit, Department of Medicine, University of Montreal Hospital Research Center (CRCHUM), Montreal, QC H2X 0A9, Canada; 3 Laboratory of Experimental Hematology, Jules Bordet Institute, Université Libre de Bruxelles, 1000 Brussels, Belgium; makram.merimi.cri@gmail.com (M.M.); Douaa.Moussa.Agha@ulb.be (D.M.A.); 4 LBBES Laboratory, Genetics and Immune Cell Therapy Unit, Faculty of Sciences, University Mohammed Premier, Oujda 60000, Morocco; 5 Gilbert and Rose-Marie Chagoury School of Medicine, Lebanese American University, P.O. Box 36, Byblos 5053, Lebanon; wissamfaour78@hotmail.com; 6 Laboratory of Pediatric Hepatology and Cell Therapy, Institut de Recherche Expérimentale et Clinique (IREC), Université Catholique de Louvain, 1200 Brussels, Belgium; calombar@gmail.com (C.A.L.); etienne.sokal@uclouvain.be (E.M.S.); 7 Orthopaedics Division, Department of Surgery, Faculty of Medicine, McGill University, Montreal General Hospital (MGH), The Research Institute of the McGill University Health Centre (RI-MUHC), Montreal, QC H3G 1A4, Canada; Yassine.Ouhaddi@gmail.com

**Keywords:** foreskin, MSCs, Th17 lymphocytes, cytokines, immunomodulation

## Abstract

Foreskin, considered a biological waste material, has been shown to be a reservoir of therapeutic cells. The immunomodulatory properties of mesenchymal stromal/stem cells (MSCs) from the foreskin (FSK-MSCs) are being evaluated in cell-based therapy for degenerative, inflammatory and autoimmune disorders. Within the injured/inflamed tissue, proinflammatory lymphocytes such as IL-17-producing T helper cells (Th17) may interact with the stromal microenvironment, including MSCs. In this context, MSCs may encounter different levels of T cells as well as specific inflammatory signals. Uncovering the cellular and molecular changes during this interplay is central for developing an efficient and safe immunotherapeutic tool. To this end, an in vitro human model of cocultures of FSK-MSCs and T cells was established. These cocultures were performed at different cell ratios in the presence of an inflammatory setting. After confirming that FSK-MSCs respond to ISCT criteria by showing a typical phenotype and multilineage potential, we evaluated by flow cytometry the expression of Th17 cell markers IL-17A, IL23 receptor and RORγt within the lymphocyte population. We also measured 15 human Th17 pathway-related cytokines. Regardless of the T cell/MSC ratio, we observed a significant increase in IL-17A expression associated with an increase in IL-23 receptor expression. Furthermore, we observed substantial modulation of IL-1β, IL-4, IL-6, IL-10, IL-17A, IL-17F, IL-21, IL-22, IL-23, IL-25, IL-31, IL-33, INF-γ, sCD40, and TNF-α secretion. These findings suggest that FSK-MSCs are receptive to their environment and modulate the T cell response accordingly. The changes within the secretome of the stromal and immune environment are likely relevant for the therapeutic effect of MSCs. FSK-MSCs represent a valuable cellular product for immunotherapeutic purposes that needs to be further clarified and developed.

## 1. Introduction

Stromal/stem cells have been the focus of intense research, introducing new possibilities for the treatment of various diseases. One of the main features of stromal/stem cell therapy is the induction of a proregenerative and tolerogenic microenvironment to promote graft acceptance and tissue repair [1]. Mesenchymal stromal/stem cells (MSCs) have attracted much interest in the fields of immunotherapy and regenerative medicine [2]. Communication between stromal and immune cells is essential to maintain tissue homeostasis and promote tissue repair. The implantation of stem cells within a milieu of inflammation will establish immediate crosstalk among stem cells, microenvironmental molecules, and resident and infiltrating immune cells [3]. As therapeutic cells, MSCs show tropism toward damaged and inflamed tissues, where they exert their beneficial effects by both direct and indirect mechanisms. MSCs have the capacity to actively sense their local molecular and cellular environment and respond by properly adjusting their regulatory machinery [2]. Indeed, several reciprocal immunobiological alterations have been reported to occur during the coculture of natural killer (NK) cells and MSCs [4]. The expression profile of immunological markers and the secretion profile of cytokines linked to immunity may influence the local inflammatory response and therefore modulate the tissue healing process [5]. A recent study reported that a bidirectional interaction between MSCs and PBMCs occurs during coculture, with a low dose of MSCs being able to stimulate PHA-activated PBMC proliferation [4]. Previous results indicated that under inflammatory circumstances, foreskin stem cells may act as specialized antigen-presenting cells [5]. Mechanistically, these functions are supported mainly by paracrine pathways [6]. Indeed, several factors play a key role in the adhesion, migration, and chemotaxis of MSCs, but the exact mechanism of homing is still unclear.

MSCs can be isolated from various tissues in the body, including bone marrow, adipose tissue, cord blood, and umbilical cord [7]. Recently, foreskin emerged as a major and important source of easy-to-reach MSCs that can be used for different medical purposes [8]. Accordingly, foreskin-derived MSCs (FSK-MSCs) were characterized as new progenitor cells with several biological properties. The isolation, expansion, and characterization of these stromal/stem cells were achieved according to the criteria of the International Society of Cellular Therapy (ISCT) [9]. Within the injured site, FSK-MSCs can modulate immune and inflammatory responses and coordinate endogenous tissue repair. In this context, they may be in the presence of proinflammatory Th17 cells known to secrete cytokines that promote inflammatory responses and contribute to the pathogenesis of multiple human autoimmune disorders [10]. Indeed, Th17 cells were found to play a key role in the etiology of a multitude of inflammatory-related diseases, including experimental autoimmune encephalomyelitis (EAE), rheumatoid arthritis (RA), and inflammatory bowel diseases (IBDs) [10,11]. As MSCs can be applied for the management of such diseases, it is therefore important to investigate the cellular and molecular interplay between FSK-MSCs and Th17 cells. Understanding how FSK-MSCs interact and modulate local immune cells within a target tissue could improve future MSC-derived therapies. Additionally, the changes within the secretome of the stromal and immune environment should be well identified for efficient therapy. Manufacturing MSCs for therapeutic purposes implies the determination of the impact of the cell ratio and inflammatory signal on their interplay with immune cells. The profile of Th17 cells and their pathway modulation by FSK-MSCs under these conditions have not been formally investigated. Herein, we investigated the in vitro impact of a low and high cell ratio in the presence of inflammation on the coculture of FSM-MSCs and activated lymphocytes. We found that in vitro cellular and molecular interplay occurs between FSK-MSCs and the Th17 cell pathway. These interactions are highly influenced by the cell ratio of the coculture as well as the presence of inflammatory signals. Depending on these conditions, FSK-MSCs altered the expression of Th17-specific markers, including interleukin-17A, interleukin-23 receptor (IL-23R), and retinoic acid receptor-related orphan nuclear receptor (ROR-γt). Moreover, FSK-MSCs differentially modulated the secretome with the coculture system, mainly by changing the profile of Th17 pathway-related cytokines (IL-1β, IL-4, IL-6, IL-10, IL-17A, IL-17F, IL-21, IL-22, IL-23, IL-25, IL-31, IL-33, INF-γ, sCD40, and TNF-α). This interplay and modulation of the Th17 pathway indicate that FSK-MSCs are sensitive and responsive and have functional plasticity. These characteristics are thus relevant to the process of tissue repair and should be used in vitro to strengthen MSCs by applying specific licensing/priming signals. Moreover, highlighting the changes within the secretome of both populations may help in the identification of mediators that may improve the therapeutic potential of FSK-MSCs in the context of an inflammatory setting.

## 2. Materials and Methods

### 2.1. Subjects

All foreskin samples were obtained from healthy male children submitted to circumcision. The research project (CE2387; 9 March 2015) was conducted according to Institut Jules Bordet ethics and recognized guidelines of the Helsinki Declaration. Informed written consent was obtained from their parents or legal guardians. The median age of the donors was 3 years (range 1–14 years old). At the time of collection, donors did not present signs of any known or visible disease or infection.

### 2.2. Isolation and Culture of FSK-MSCs

Foreskin samples (*n* = 7) were aseptically collected after circumcision into a sterile 50 mL conical sterile flask filled with sterile warm (1×) phosphate-buffered saline (PBS; Lonza, Braine l’Alleud, Belguim) buffer supplemented with penicillin/streptomycin. The samples were processed as previously described [11]. Briefly, after a sterile wash with warm (1×) PBS, the sample was transferred into a Petri dish, the epidermis was surgically removed from the skin, and the remaining dermis was minced into small pieces. Minced tissue was digested with Liberase Research Grade solution (Roche Diagnostics, Vilvoorde, Belgium), and the cell suspension was washed by centrifugation (800× *g*, 5 min) in Dulbecco’s modified Eagle’s medium with low glucose (DMEM-LG; Lonza, Belguim) containing 10% fetal bovine serum (FBS; Sigma–Aldrich, Diegem, Belgium). We collected the cell pellet and seeded it in culture flasks with DMEM-LG (Lonza) supplemented with 10% FBS (Sigma–Aldrich), 2 mM l-glutamine, and 50 U/mL penicillin (both from Lonza), and incubated at 37 °C in a 5% CO_2_ humidified atmosphere. By changing the medium (once a week), nonadherent cells were removed. When subconfluence (80–90%) was achieved, adherent cells were harvested by TrypLE Select (Lonza) and expanded until passage 2.

### 2.3. Characterization of FSK-MSCs

FSK-MSCs were characterized according to ISCT standards by establishing their phenotype and their differentiation potential [12]. The immunophenotype of human FSK-MSCs was established by flow cytometry using the monoclonal antibodies indicated in Table 1. Adherent cells were harvested with TrypLE Select (Lonza) solution, washed by centrifugation in PBS (Lonza), and finally resuspended in Miltenyi Biotec buffer. Then, harvested cells were incubated for 30 min at room temperature (RT) with conjugated primary antibody. After the labeling period, the cells were again washed, resuspended in PBS, and fixed with % paraformaldehyde. After cell staining, data were acquired and analyzed on a MacsQuant analyzer (Miltenyi Biotec, Leiden, The Netherlands). The differentiation potential of FSK-MSCs was confirmed by inducing differentiation into adipogenic, osteogenic, and chondrogenic lineages using the appropriate culture conditions (NH media, Miltenyi Biotec) as previously described [13].

### 2.4. In Vitro Differentiation Assay

#### 2.4.1. Osteogenic Differentiation

A total of 5000 cells/well were plated in a 24-well plate with culture medium. After 5 days, the medium was removed and replaced with osteogenic medium (StemMACS Osteo-Diff Media, Miltenyi Biotec). Cells were fed weekly with complete replacement of osteogenic medium. After 21 days, the mineralization of the extracellular matrix was assessed by Alizarin red staining. Cells were washed in phosphate-buffered saline (PBS) and fixed in 70% ethanol at room temperature for 5 min, followed by several washes in H_2_O. Cells were stained in 40 mM Alizarin red (Sigma–Aldrich) pH = 4.2 for 15 min at room temperature, rinsed in H_2_O, and then air-dried. The red staining was examined by light microscopy.

#### 2.4.2. Adipogenic Differentiation

A total of 5000 cells/well were plated in a 24-well plate with culture medium. After 5 days, the medium was removed and replaced with adipogenic medium (StemMACS AdipoDiff Media, Miltenyi Biotec). Cells were fed weekly with complete replacement of adipogenic medium. On day 7, cells were stained with Oil Red O solution (Sigma) after fixation (8% formaldehyde). Lipid vacuoles were then observed by light microscopy.

#### 2.4.3. Chondrogenic Differentiation

A total of 150,000 cells were cultured in the tip of a 15 mL conical tube (Greiner, Vilvoorde, Belgium) to enable cell culture in micromass with chondrogenic medium (Stem MACS Chondro Diff Media, Miltenyi Biotec). Cells were resuspended carefully and cultured at 37 °C and 5% CO_2_ in a humidified atmosphere with a slightly screwed cap. Half of the chondrogenic medium was replaced weekly. On day 21, aggregates were stained with Alcian blue (Sigma–Aldrich, Diegem, Belgium) to highlight cartilage proteoglycans. In some cases, cryosectioned pellets were stained with Alcian blue to confirm chondrogenic differentiation.

### 2.5. Inflammatory Priming of FSK-MSCs

Priming of the MSCs was performed as described in our previous publication. We evaluated the impact of an inflammatory environment on FSK-MSC expression of immune-associated molecules and factors. Tissue-derived FSK-MSCs were primed overnight using a cocktail of proinflammatory cytokines, specifically 25 ng/mL IL-1β (Peprotech, Rocky Hill, NJ, USA) and 103 U/mL IFN-γ, 50 ng/mL TNF-α, and 3 × 103 U/m IFN-α (all from Peprotech, Rocky Hill, NJ, USA). After priming, MSCs were analyzed by flow cytometry.

### 2.6. Immunomodulation and Coculture Assays

Immunomodulation assays were performed as previously described [14]. Briefly, peripheral blood mononuclear cells (PBMC) were obtained by Ficoll-Hypaque gradient centrifugation of peripheral blood from healthy donors with informed consent. Purification of T lymphocytes (>95% purity) was performed by positive selection using the MACS system (Miltenyi Biotec). A cocktail of phytohemagglutinin (PHA, 5 μg/mL; Remel Europe, Kent, UK) and interleukin-2 (IL-2, 20 U/mL; Biotest AG, Dreieich, Germany) was used to activate T lymphocytes. Activated T cells and stimulated or unstimulated FSK-MSCs were plated in cocultures at both cell ratios: 1/80 and 1/5 for 5 days.

### 2.7. Human Th17 Characterization

The modulation of Th17 cells was assessed by flow cytometry analysis by identifying and characterizing three main markers: IL-17A, ROR-γt, and IL-23R.

### 2.8. IL-17A Expression

IL-17A expression was measured by flow cytometry using a human IL-17A APC-conjugated antibody, according to the manufacturer’s protocol (R&D Biosystems).

### 2.9. RORγt Expression

RORγt expression was measured by flow cytometry using a human RORγt FITC-conjugated antibody, according to the manufacturer’s protocol (R&D Biosystems).

### 2.10. IL-23 Receptor Expression

IL-23 receptor (IL-23R) expression was measured by flow cytometry using a human IL-23R PE-conjugated antibody according to the manufacturer’s protocol (R&D Biosystems).

### 2.11. Th17 Cytokine Pathway

By using the Bio-Plex Pro™ Human Th17 Cytokine Assays^®^ (Bio–Rad Laboratories, Inc., Temse, Belgium), the concentrations of multiple cytokines involved in the immunobiology of Th17 were determined according to the manufacturer’s guidelines. The multiplex format enables robust and reproducible measurement of the following cytokines (IL-1β, IL-4, IL-6, IL-10, IL-17A, IL-17F, IL-21, IL-22, IL-23, IL-25, IL-31, IL-33, IFNγ, soluble CD40 ligand (sCD40L), and TNFα) from different culture supernatants. 

### 2.12. Experimental Conditions

- MSCs at two different cell ratios were used: 1/80 (low MSCs) and 1/5 (high MSCs).
- MSCs were or were not primed with inflammation.
- MSCs were or were not cultured with activated T cells.
- T cells activated or not were used as controls.

### 2.13. Flow Cytometry

The data were acquired and analyzed on a MacsQuant analyzer (Miltenyi Biotec, Leiden, The Netherlands).

### 2.14. Statistics

Data are expressed as the mean ± standard error of the mean. Statistical analysis was performed using Prism software for paired samples. Statistical significance was measured by ANOVA for repeated measures followed by a Newman–Keuls multiple comparison test. A value of * *p* < 0.05 was considered statistically significant, ** *p* < 0.01 or *** *p* < 0.001.

## 3. Results

**Figure 1:**
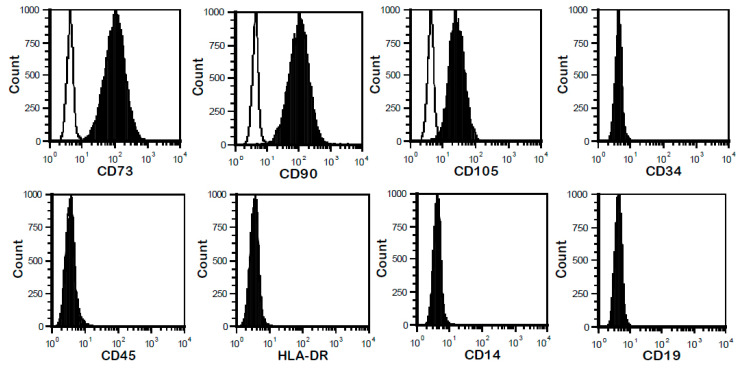
Flow cytometry profile of FSK-MSCs. Human FSK-MSCs were stained with specific fluorochrome-labeled monoclonal antibodies (filled histograms) against CD14, CD19, CD34, CD45, HLA-DR, CD73, CD90, and CD105. In each image, empty histograms show the background staining with the isotype control (control staining), and solid black histograms represent the specific staining for the indicated cell surface markers. For each analysis, the marker is given under the plot.

FSK-MSC characterization. The properties of isolated FSK-MSCs were characterized according to the criteria of the International Society for Cellular Therapy (ISCT). These cells showed a fibroblastic shape and adhered to plastic during culture. Regarding their phenotype, a representative flow cytometry profile is presented (Figure 1). FSK-MSCs expressed mesenchymal stromal/stem cell markers CD73, CD90, and CD105, but they did not express hematopoietic stem cell markers, including CD45, CD14, CD19, CD34, and HLA-Dr. Table 2 shows the different percentages of expression for each marker as determined by flow cytometry.

**Figure 2:**
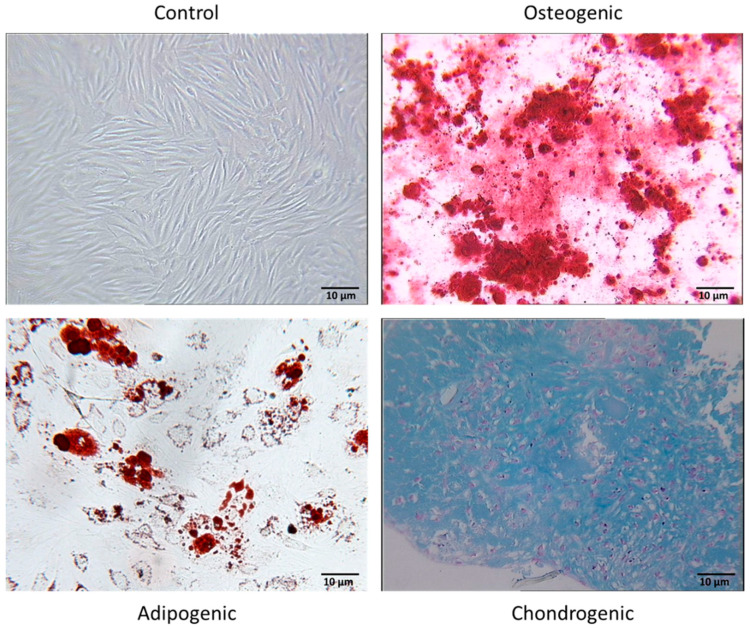
The multilineage potential of FSK-MSCs. The in vitro differentiation profile of human FSK-MSCs was assessed by using special induction medium and staining techniques (magnification 100×). A representative example of osteogenesis from FSK-MSCs was evaluated after 21 days of induction by Alizarin red staining to color calcium deposits (mineralization). A representative example of adipogenesis from FSK-MSCs was evaluated after 7 days of induction by Oil Red O staining to color lipid vacuole formation. A representative example of chondrogenesis from FSK-MSCs was evaluated after 21 days of induction by Alcian blue staining to color extracellular matrix (proteoglycans).

The culture of the cells in the presence of special induction medium and by using lineage-specific cell staining techniques allowed us to observe the in vitro multilineage potential of FSK-MSCs (Figure 2). FSK-MSCs were able to undergo osteogenesis, as shown by matrix mineralization following calcium deposition (Alizarin red staining). Adipogenesis was induced as adipocytes were generated, and their cytoplasm contained a high number of small lipids (Oil Red O solution). Chondrogenesis, as the production of a proteoglycan-based extracellular matrix, was achieved and was shown by Alcian blue staining.

### 3.1. Th17 Proportion in Coculture with FSK-MSCs

**Figure 3:**
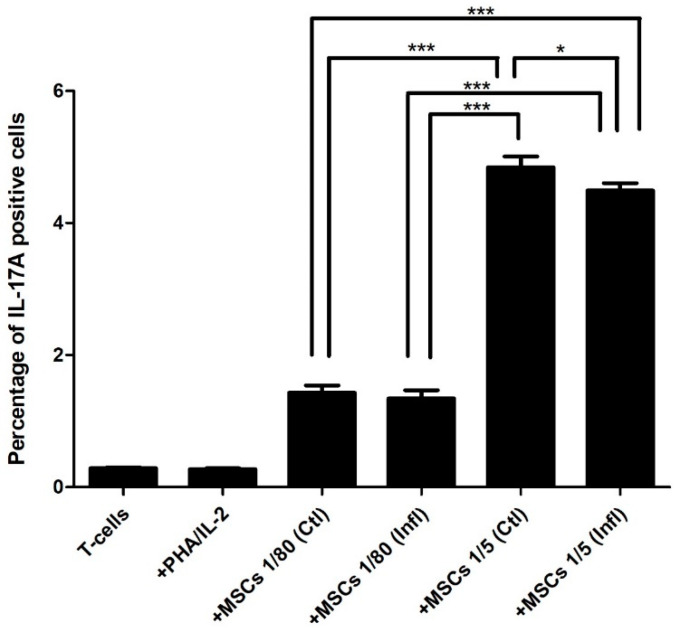
Influence of FSK-MSCs on IL-17A. FSK-MSCs were cocultured at a (1:5) or (1:80) cell ratio under normal and inflammatory conditions with either unactivated or PHA/IL-2-activated T cells as indicated. Levels of IL-17A expression were analyzed by flow cytometry. Data are expressed as the percentage of IL-17A ± SEM-positive cells from seven independent experiments. * *p* < 0.05, *** *p* < 0.01 versus control.

Flow cytometry analysis showed that the proportion of Th17 cells was not induced in stimulated T cells (0.271666 ± 0.0343) but was significantly increased in cocultures at a 1:80 ratio (1.431666 ± 0.2700) compared to the proportion of Th17 cells in unstimulated T cells (0.285 ± 0.0437) alone. Additionally, the ratio of T cells to MSCs in the coculture had a great impact on the Th17 proportion, as a 1:5 ratio (4.84333 ± 0.4029) showed a significant increase in the Th17 proportion compared to T cell/MSC cocultures at a 1:80 ratio (1.431666 ± 0.2700). Of note, inflammation had no effect on the Th17 proportion in cocultures at either the 1:80 (1.34166 ± 0.3145) or 1:5 ratio (4.49333 ± 0.2690) when compared to cocultures at the 1:80 and 1:5 ratios not exposed to inflammation (1.431666 ± 0.2700; 4.84333 ± 0.4029), respectively (Figure 3).

### 3.2. Th17-Associated ROR-γt Expression during Coculture with FSK-MSCs

**Figure 4:**
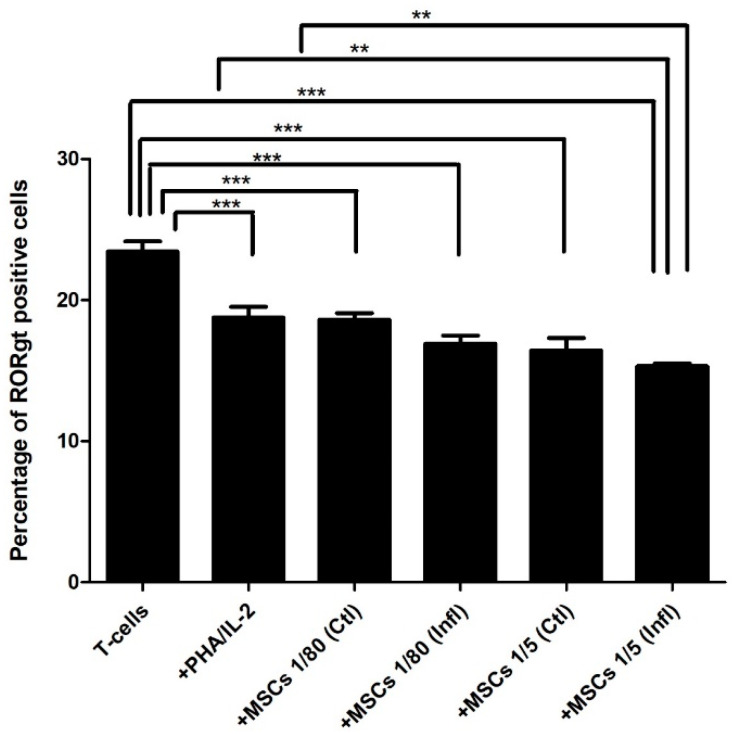
Influence of FSK-MSCs on ROR-γt expression. FSK-MSCs were cocultured at a (1:5) or (1:80) cell ratio under normal and inflammatory conditions with either unactivated or PHA/IL-2-activated T cells as indicated. Levels of ROR-γt expression were analyzed by flow cytometry. Data are expressed as the percentage of RORgt ± SEM-positive cells from seven independent experiments. ** *p* < 0.01, *** *p* < 0.01 versus control.

Our results showed that in stimulated T cells, RORγt expression (18.765 ± 1.8889) was reduced compared to RORγt expression levels in unstimulated T cells (23.4433 ± 1.7485). Interestingly, T cell/MSC cocultures at both 1:80 and 1:5 ratios either in the presence (16.9116 ± 1.4054; 15.32 ± 0.50966) or absence (18.625 ± 1.1297; 16.435 ± 2.1971) of inflammation showed lower RORγt expression levels than both unstimulated T cells (23.4433 ± 1.7485) and stimulated T cells (18.765 ± 1.8889) (Figure 4).

### 3.3. Th17-Associated IL-23 Receptor Expression during Coculture with FSK-MSCs

**Figure 5:**
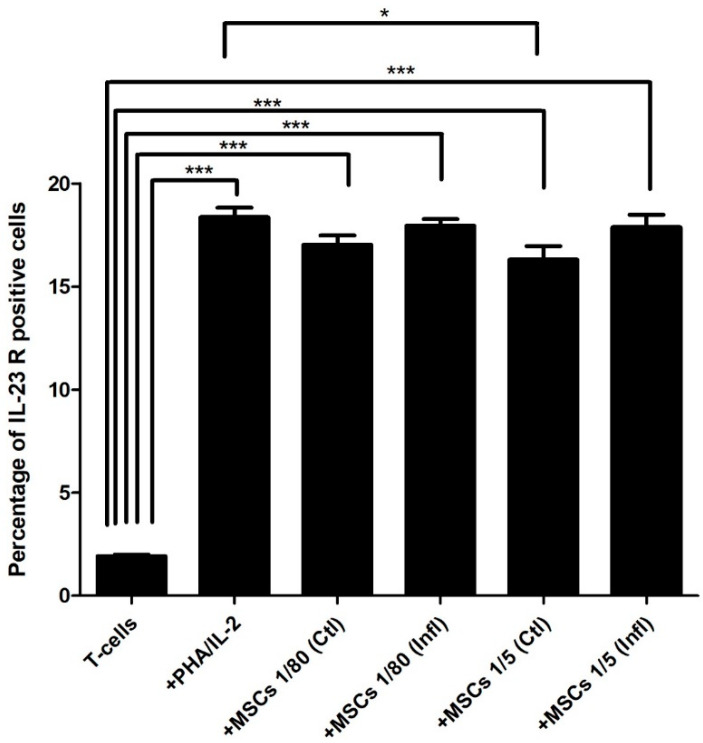
Influence of FSK-MSCs on IL-23 receptor expression. FSK-MSCs were cocultured at a (1:5) or (1:80) cell ratio under normal and inflammatory conditions with either unactivated or PHA/IL-2-activated T cells as indicated. Levels of IL-23 receptor expression were analyzed by flow cytometry. Data are expressed as the percentage of IL-23 receptor ± SEM-positive cells from seven independent experiments. * *p* < 0.05, *** *p* < 0.01 versus control.

Interestingly, stimulated T cells (18.36 ± 1.137) showed significantly elevated IL-23R expression levels compared to expression levels in unstimulated T cells (1.922 ± 0.1628). Interestingly, IL-23R expression levels in cocultures at both 1:80 and 1:5 ratios of T cells with either inflammatory primed FSK-MSCs (17.96 ± 0.7753; 17.87 ± 1.499) or unstimulated FSK-MSCs (17.03 ± 1.097; 16.31 ± 1.574), respectively, were significantly higher than IL-23R expression levels in unstimulated T cells alone (1.922 ± 0.1628) (Figure 5).

### 3.4. Th17 Associated Cytokine Profile

Our data showed that stimulated T cells, when compared to unstimulated T cells, produced significantly higher amounts of IL-1β (17.67 ± 1.90; 0.46 ± 0.12), IL-6 (696.6625 ± 51.6438919; 8.23 ± 0.42720019), IL-17A (280.91 ± 36.18; 4.55 ± 0.85), IL-17F (337.92 ± 21.75; 8.18 ± 0.84), IL-21 (251.41 ± 8.06; 36.43 ± 2.38), IL-22 (179.61 ± 6.25; 8.35 ± 0.98), IL-23 (103.89 ± 3.77; 53.53 ± 5.34), IL-31 (716.21 ± 2.40; 5.19 ± 0.24), INF-γ (4458.20 ± 317.75; 24.04 ± 3.76), sCD40 (871.49 ± 40.18.915 ± 10.917), and TNF-α (1537.915 ± 52.20 ± 317.75; 24.04 ± 1461445). While unstimulated T cells produced negligible amounts of IL-4, IL-10, IL25, and IL-33, stimulated T cells produced very low levels of IL-25 (1.51 ± 0.31), detectable levels of IL-33 (11. 5075 ± 2. 40701449), and significantly higher levels of IL-4 (522.285 ± 18.6969911) and IL-10 (290.33 ± 13.28).

Cocultures of unstimulated T cells with FSK-MSCs at both 1:80 and 1:5 ratios produced negligible to very low amounts of all the abovementioned cytokines except for the cytokines IL-6 (1683.93 ± 195.07; 3062.55 ± 584.42), IL-17F (18.76 ± 0.77; 43.24 ± 4.83), IL-21 (22.66 ± 5.05; 118.91 ± 2.40), IL-23 (49.08 ± 2.64; 40.27 ± 3.59), and sCD40 (9.35 ± 1.02; 60.81 ± 4.52). Interestingly, cocultures of T cells with inflammatory primed MSCs at both 1:80 and 1:5 ratios, respectively, showed significantly higher levels only of the following cytokines: IL-1β (84.42 ± 2.40; 152.97 ± 5.94), IL-6 (23822.49 ± 1167.89; 25081.34 ± 0.00), IL-17F (64.43 ± 5.75; 69.10 ± 5.9), IL-21 (263.47 ± 11.57; 328.05 ± 12.53), IL-23 (122.27 ± 3.86; 107.22 ± 1.24), IL-31 (244.80 ± 6.60; 257.62 ± 13.12), INF-γ (2221.15 ± 395.56; 1563.03 ± 43.16), sCD40 (84.63 ± 2.94; 97.53 ± 3.72), and TNF-α (444.12 ± 30.87; 287.27 ± 5.93). Meanwhile, levels of the cytokines IL-4 (8.24 ± 1.23; 8.12 ± 0.21), IL-10 (8.76 ± 1.09; 6.75 ± 1.22), IL-17A (7.12 ± 0.97; 7.60 ± 0.85), IL-22 (0.00; 0.00), IL-25 (2.19 ± 0.17; 2.27 ± 0.19), and IL-33 (0.00; 0.00) were very low and comparable to levels found in cocultures of Tcells/MSCs at both 1:80 and 1:5 ratio not exposed to inflammation. Furthermore, cocultures at a 1:80 ratio of either stimulated T cells with MSCs or stimulated T cells with inflammatory primed FSK-MSCs showed, respectively, significant increases in the amounts of IL-1β (74.32 ± 4.21; 184.75 ± 4.19), IL-6 (24861.54 ± 439.61; 23782.88 ± 1022.02), IL-21 (467.18 ± 20.45; 458.49 ± 9.46), IL-23 (140.15 ± 1.88; 153.82 ± 3.05), and IL-17A (454.55 ± 16.90; 451.53 ± 22.10). In turn, the levels of IL-4 (213.12 ± 3.34; 146.97 ± 5.82), IL-10 (148.10 ± 10.34; 235.87 ± 13.76), IL-17F (167.21 ± 34.22; 129.21 ± 33.29), IL-22 (87.08 ± 6.53; 65.77 ± 3.47), and TNF-α (791.03 ± 58.13; 1240.58 ± 32.15) were significantly reduced when compared to levels in stimulated T cells. Of note, the levels of IL-31 (613.52 ± 4.35; 777.34 ± 92.88), INF-γ (3639.93 ± 454.37; 5967.38 ± 800.06), and sCD40 (677.19 ± 140.12; 686.25 ± 12.67) remained comparable to the levels in stimulated T cells. Finally, the levels of IL-25 and IL-33 were found to be negligible. Regarding cocultures of unstimulated T cells with FSK-MSCs at a 1:5 ratio, when these cocultures were compared to unstimulated T cells alone, they showed negligible IL-4, IL-10, IL-17A, IL-22, IL-25, IL-33, and TNF-α and very low levels of IL-1β (0.53 ± 0.18), IL-23 (40.27 ± 3.59), IL-25 (1.69 ± 0.37), IL-31 (14.26 ± 2.4), and INF-γ (24.26 ± 3.69) but significantly higher levels of IL-6 (3062.55 ± 584.42), IL-17F (43.24 ± 4.83), IL-21 (118.91 ± 2.40), and sCD40 (60.81 ± 4.52). In cocultures of unstimulated T cells with inflammatory primed FSK-MSCs at a 1:5 ratio, the levels of IL-4 (8.12 ± 0.21), IL-10 (6.75 ± 1.22), IL-17A (7.60 ± 0.85), IL-22 (0.00 ± 0.00), IL-25 (2.27 ± 0.19), and IL-33 (0.00 ± 0.00) remained very low, but the levels of IL-1β (152.97 ± 5.94), IL-6 (25081.34 ± 0.00), IL-17F (69.10 ± 5.90), IL-21 (328.05 ± 12.53), IL-23 (107.22 ± 1.24), IL-31 (257.62 ± 13.12), INF-γ (1563.03 ± 43.16), sCD40 (97.53 ± 3.72), and TNF-α (287.27 ± 5.93) were significantly increased when compared to unstimulated T cells alone or to cocultures of unstimulated T cells with FSK-MSCs not exposed to inflammation.

**Figure 6:**
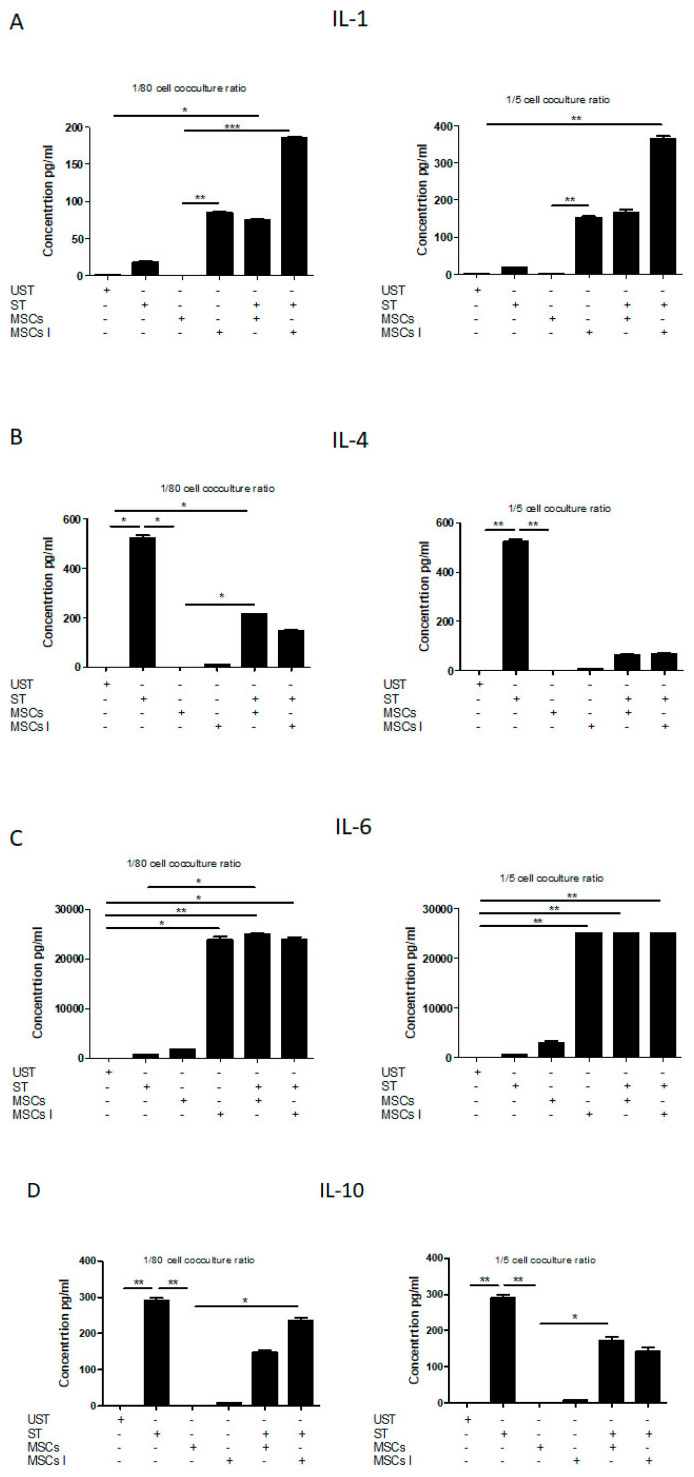

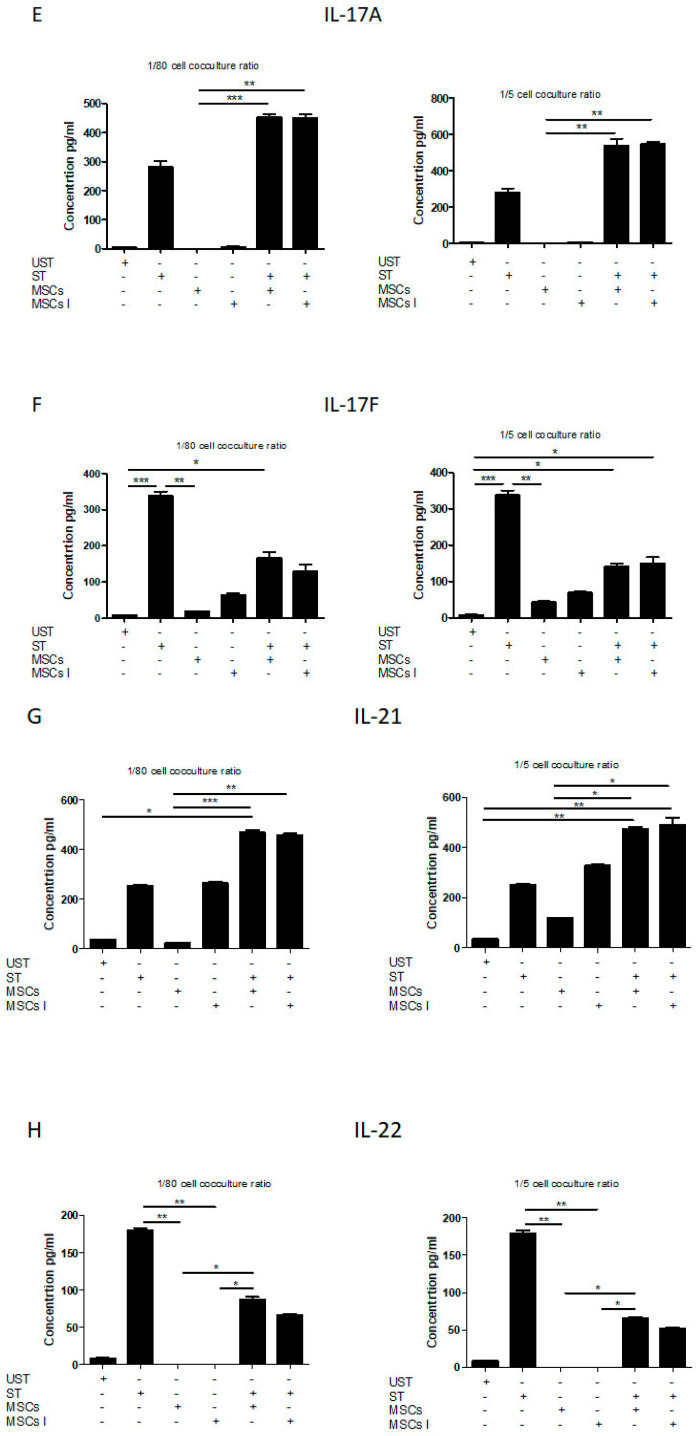

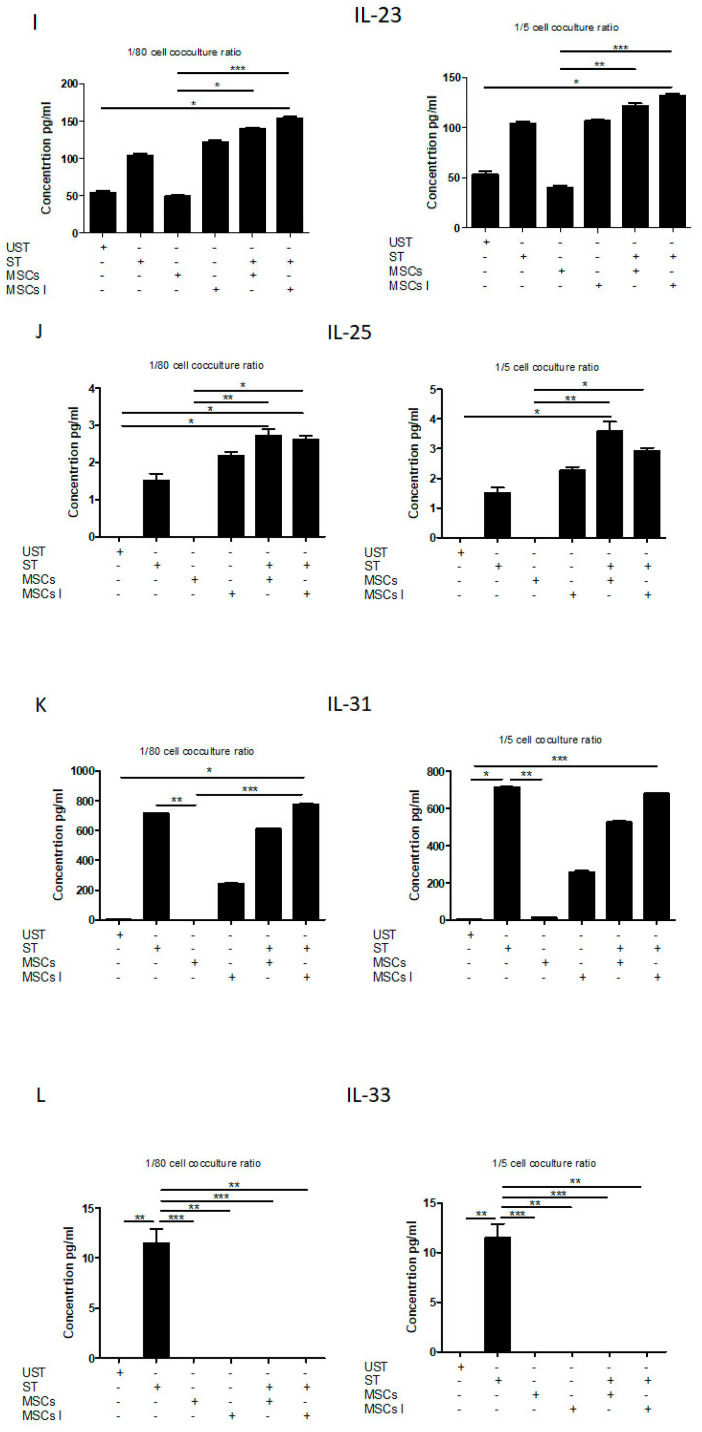

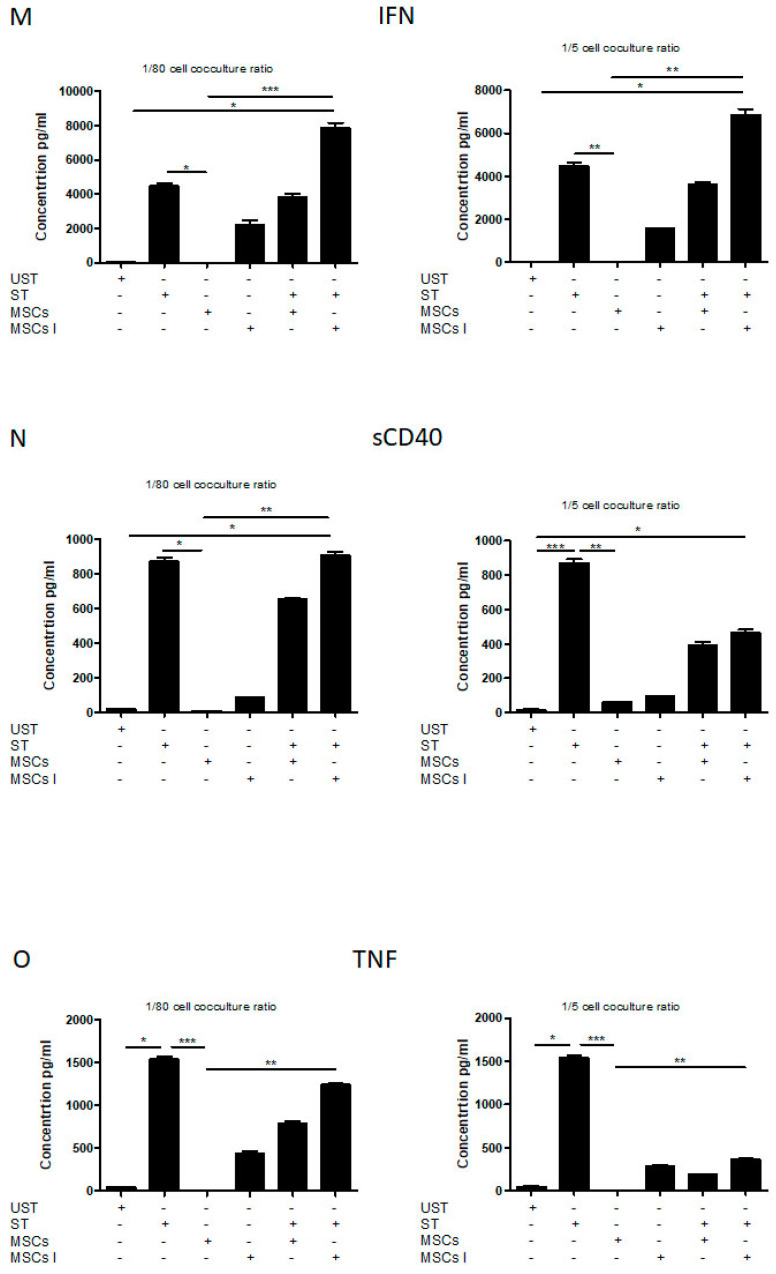
Th17-associated cytokine array profile. FSK-MSCs were cocultured at a (1:5) or (1:80) cell ratio under normal and inflammatory conditions with either unactivated or PHA/IL-2-activated T cells as indicated. The levels of the different cytokines were analyzed as indicated in the Materials and Methods section. Data are presented as the concentration ± SEM (pg/mL) for IL-1β (**A**), IL-4 (**B**), IL-6 (**C**), IL-10 (**D**), IL-17A (**E**), IL-17F (**F**), IL-21 (**G**), IL-22 (**H**), IL-23 (**I**), IL-25 (**J**), IL-31 (**K**), IL-33 (**L**), INF-γ (**M**), sCD40 (**N**), and TNF-α (**O**) from seven independent experiments. * *p* < 0.05, ** *p* < 0.01, *** *p* < 0.01 versus the corresponding control. UST: unstimulated lymphocytes; FSK-MSC: foreskin-derived mesenchymal stromal cell; IL: interleukin; PHA: phytohemagglutinin; ST lymph: PHA/IL-2 stimulated lymphocytes; FSKi MSC: inflammatory primed MSCs.

Finally, the cocultures at a 1:5 ratio of stimulated T cells with either FSK-MSCs or inflammatory primed FSK-MSCs showed significantly higher production levels of IL-1β (164.90 ± 16.88; 365.61 ± 11.10), IL-6 (25081.34 ± 0.00; 25081.34 ± 0.00), IL-17A (538.33 ± 74.09; 546.32 ± 20.48), IL-21 (475.91 ± 14.22; 492.75 ± 45.88), and IL-23 (121.47 ± 5.41; 131.59 ± 3.90) than unstimulated T cell and FSK-MSC cocultures at both 1:80 and 1:5 ratios, as well as stimulated T cells alone. Interestingly, they both showed significantly lower levels of IL-4 (62.73 ± 9.68; 66.62 ± 7.49), IL-10 (172.62 ± 19.84; 142.99 ± 19.53), IL-17F (140.40 ± 18.66; 149.86 ± 29.30), IL-22 (65.75 ± 3.52; 51.20 ± 2.77), IL-31 (527.25 ± 15.49; 680.93 ± 5.21), sCD40 (394.52 ± 32.76; 463.16 ± 37.14), and TNF-α (182.34 ± 11.71; 363.26 ± 14.51) than stimulated T lymphocytes. Additionally, the levels of IL-25 (3.59 ± 0.64; 2.92 ± 0.18) remained very low and negligible for IL-33 in both cocultures of stimulated T cells and FSK-MSCs when compared to stimulated T cells alone. INF-γ levels remained almost unchanged in cocultures of stimulated T cells with FSK-MSCs (3611.42 ± 263.80) but increased in cocultures of stimulated T cells with inflammatory MSCs (6865.21 ± 430.45) (Figure 6).

## 4. Discussion

The regenerative properties of stromal/stem cells have fueled research interest toward their use with the hope of treating devastating chronic illnesses. Stromal/stem cell interactions with either resident or infiltrating immune cells are a key event in determining the success of the therapy (https://pubmed.ncbi.nlm.nih.gov/34490235/). The implantation of these cells in a site of inflammation will establish changes within the cellular and molecular profile of the microenvironment (https://pubmed.ncbi.nlm.nih.gov/34298927/). Accordingly, the quality and safety of MSC transplantation requires appropriate analysis and characterization of their immune–inflammatory interplay (https://pubmed.ncbi.nlm.nih.gov/33404674/).

Human foreskin provides an interesting source of MSCs for immunotherapy purposes that comply with the ISCT criteria [7]. Indeed, they presented in vitro multilineage capacities by showing adipogenic, osteogenic, and chondrogenic differentiation following culture using an appropriate induction medium. Although MSCs derived from different tissues may share the same in vitro trilineage differentiation potential, some differences can be observed between the sources of MSCs [15,16]. In general, no differences are observed among samples derived from the same source [17]. The phenotype of FSK-MSCs was characterized by constitutively high expression of CD73, CD90, and CD105 and a lack of CD14, CD19, CD34, and CD45. Specifically, no significant differences were reported among the different samples for the expression of these markers. In particular, we observed a slight difference in the expression of CD105 among the different samples. The expression of these markers by tissue-specific MSCs can be clearly different. Although stable expression of CD73 and CD90 is commonly described, the expression level of CD105 can vary significantly among MSCs from different sources [18]. For some authors, there are some inconsistent data about the suitability of CD105 as a typical MSC marker [19]. The expression of CD105, as a usual surface marker of MSCs, changed in AFMSCs grown with different protocols, but this change was not associated with human amniotic fluid mesenchymal stem cell (AFMSC) biological function [20]. Due to the broad variety of potential tissue sources and the differences in cell isolation and cell culture procedures used, some differences within the literature may be raised. In addition, differences in media formulations (FBS, platelet lysates, growth factor combinations, etc.), plating density, and oxygen tension may affect the phenotype of the mesenchymal population [21]. Thus, a study described that the characteristics of human BM-MSCs can be maintained during culture expansion and that a homogeneous phenotype of undifferentiated MSCs persists independent of cell density [22], whereas another study indicated that human BM-MSCs subjected to extensive in vitro passage can undergo phenotypic changes [23]. However, the ISCT and IFATS regulatory committees maintain the expression of CD105 as a marker to define MSCs [24].

The specific culture conditions utilized in this study may have influenced the results obtained. Due to their limited number after isolation, in vitro culture and expansion are required to achieve a sufficient number of cells for clinical and research applications. In particular, the culture medium composition (serum, antibiotic/antimycotic, and growth factors), well-bottom surface (affect cell adherence), cell ratio in cocultures (affect cellular contact), responder cell type (total PBMCs, splenocytes, purified T cells), MSC origin (murine or human), source (bone marrow, cord, placental, adipose tissue), age, sex, and health status of the donors (donor variability), and tissue environment challenges (inflammation, hypoxia, infection) may also affect the outcome of the study [25]. Of importance, the immunomodulatory capacity of MSCs is dependent on the type and level of inflammatory mediators within their microenvironment. In fact, different states of inflammation can result in significantly different responses of MSCs, indicating the plasticity of their immunomodulation [26,27]. Despite their prolific use in therapy, sex-specific mechanisms of action are rarely considered potential confounding factors for the use of MSCs. However, a study recently highlighted that female MSCs are more potent in vitro than their male counterparts for modulating the immune response [28]. Chromosomal segments and differentially expressed genes in male and female adipose MSCs were related to several processes, such as inflammation, differentiation, and cell communication. Such results lead to the hypothesis that the donor sex of MSCs is a variable influencing a wide range of stem cell biologic processes [29].

Since there is yet no medium that is as close to natural in vivo fluids to support physiological MSC culture, specialized culture media are composed based on the cell type, type of culture, and focus of analysis. Additional focus is placed on the effects of other cell culture variables, including physiochemical conditions and subculture protocols. Therefore, it is necessary to define what constitutes an “optimal practice” for maintaining the physiological status of MSCs during their primary step of in vitro 2D expansion. The influence of antibiotics on the biology of MSCs should also not be neglected. Indeed, penicillin, streptomycin, amphotericin B, and AmB-Cu2+ have been shown to alter the proliferation, viability, and differentiation potential of MSCs [30]. Recent results have also indicated the extent to which the type of serum (as an essential component for cell cultivation) and its modification are capable of affecting the biology and behavior of MSCs. Choosing a suitable FBS percentage is a controversial issue and depends merely on the experience of the researchers and the recommendations suggested by the suppliers [31]. Several differences in the sensitivity of human MSCs to various sera and their alternatives have been discussed [32]. Several changes in the morphology, phenotype, metabolism, and cytokine production of MSCs cultivated in the presence of these compounds have been reported. The authors revealed a significant impact of the heat inactivation of FBS on MSC metabolism and CFU-F efficiency. Data confirmed that AB serum and platelet releasates provide efficient and easily accessible alternatives to FBS for MSC cultivation purposes (the analysis suggested that they are comparable to or more effective than FBS). Although FBS is a typical serum widely used in the field of MSCs, no batch is the same in terms of composition and quality, and FBS is chemically undefined, which is detrimental with regard to experimental reproducibility [32]. It is therefore the time to accelerate the transition from the use of xenogeneic FBS to human serum alternatives. Human serum and blood platelet derivatives are currently promising and relatively well-accessible alternatives [32]. The origin of the serum and its heat inactivation may critically influence the behavior of MSCs. Despite their batch-to-batch variability, these alternatives represent promising supplements due to their human, native, accessible, and ethical origin; further detailed analyses and studies will be required, and guidelines will have to be set to fully guarantee the safety and efficiency of these alternatives. In some circumstances, the price of these commercial alternatives may sometimes be prohibitive [32]. Producing MSCs for clinical use requires adherence to current good manufacturing practice (cGMP) standards, which is necessary not only for ensuring standardization and reproducibility through the manufacturing process but also for product quality and safety. The quality and safety are of importance for therapeutic purposes, especially because of the numerous variations in preclinical and clinical protocols for MSC-based products [33]. Chemically well-defined xenogeneic-free media supporting the growth of MSCs would constitute a more cost-effective and risk-reduced approach. Although these new formulations may influence MSC phenotype and functions, they will have the potential to enhance batch-to-batch consistency [34]. The GMP manufacturing of MSC products is feasible in the academic setting for a limited number of batches with a significant cost increase, but moving to the large-scale production necessary for phase III trials would require the involvement of industrial partners [35]. Several systems, such as the CliniMACS Prodigy^®^ [36], serum-free, xeno-free, and chemically defined medium (S&XFM-CD) [37], have been developed and may serve as a clinical-grade production platform for MSCs. To our knowledge, there are currently no other publications investigating the use of foreskin as a source of therapeutic MSCs complying with ISCT or considering the standardization of the clinical-grade production of FSK-MSCs. At the Sidra Medicine GMP facility, FSK-MSCs are being developed according to ISCT criteria for several clinical applications [38].

Furthermore, the preservation of the physiological characteristics of MSCs during their in vitro culture is essential for improving the efficiency of therapeutic and in vitro modeling applications, as recently reviewed [39]. In view of this document, there is considerable research focus on the optimization of specific culture media, culture conditions, and protocols for MSCs. The development should focus on culture media components as well as culture conditions and techniques to preserve the fate of MSCs.

MSCs are defined as environmentally responsive cells because they are capable of responding to local surrounding stimuli with a myriad of beneficial interventions [40]. These cells are specifically home to damaged and inflamed tissues and contribute to their repair in part by secreting immunomodulating cytokines, chemokines, and extracellular matrix proteins [41]. The development of an inflammatory environment follows any tissue injury, and MSCs may be exposed to such stimuli under many clinical conditions. As an example, inflammation is known to be a major event triggering graft-versus-host disease (GVHD) after allogeneic stem cell transplantation [42]. Thus, inflammation is described to critically modulate the properties of stem cells during infection and beyond [43]. Several studies have shown that tissue-derived MSCs are differentially sensitive to inflammation [44,45,46]. To mimic inflammation, a cocktail of proinflammatory cytokines is frequently used, as these cytokines are mostly present at inflammatory sites, where they act together to modulate different cellular processes [47]. Indeed, under inflammatory conditions, high levels of cytokines such as IFNγ/α, TNF-α, and IL-1-β are largely reached. Rapid, intense exposure to high concentrations of proinflammatory cytokines can produce very different cellular responses compared to longer exposure and lower concentrations. In the majority of studies evaluating the impact of inflammation on cell biology, the concentrations of proinflammatory cytokines were very similar to those used in our study [48,49,50]. Our group and several others have reported that overnight preincubation of MSCs with this proinflammatory cytokine cocktail is efficient in modulating the biology and functions of MSCs. Indeed, these cytokines are known to cooperate in regulating the transcription of a large number of genes important to inflammation and immunoregulation [51,52,53]. We have thus demonstrated that priming MSCs with inflammation altered their expression of cellular adhesion molecules [54], changed their immunological profile [18,55], modified their pattern of TLR [56], and finally affected their differentiation potential [57,58]. Of importance, the immunomodulatory ability of MSCs is dependent upon the kinds and concentrations of inflammatory mediators present in their microenvironment. In fact, different states of inflammation can result in markedly different responses to MSC treatment, which indicates the plasticity of immunomodulation by MSCs [26].

Based on this evidence, the results regarding the modulation of Th17 cells by MSCs have been either sparsely discussed or influenced by the culture conditions and methodology. Thus, further understanding and evaluation of the different features that could affect such interactions should be investigated. The mechanism by which MSCs exert their regulatory effects in vivo remains largely unknown, thus limiting both the general interest and the clinical impact of these studies [59]. The importance of this study arises from the detrimental role that Th17 cells play in many autoimmune and inflammatory diseases [60] and where MSCs might be proposed as a therapeutic strategy. Moreover, multiple factors linked to MSCs, immune cells, and the local environment may significantly influence the therapeutic effects [61]. Th17 cells are greatly influenced by the presence of MSCs and cytokines that determine their polarization status and therefore their immunological features. The balance of graft-protective regulatory and graft-destructive effector T cells depends largely on the balance of proinflammatory and anti-inflammatory cytokines in the milieu in which the donor-directed T cell response occurs [62]. A better understanding of the interplay between engrafted MSCs and Th17 cells is needed to optimize the therapeutic issue. Th17 subsets do not represent stable differentiation processes and display a great degree of context-dependent plasticity. During tissue injury, a plethora of biological factors, including growth factors, cytokines, and chemokines released near the stromal/stem cell niche, as well as ongoing immune and inflammatory processes, will certainly influence the outcome of the interactions between MSCs and T cells and therefore have a critical impact on the healing of damaged tissue. Moreover, a high MSC:T cell ratio significantly suppressed T cell activation, while a low cell ratio distinctively stimulated T cells. Interestingly, we have previously found that FSK-MSCs interact and modulate several functions of NK cells [23]. Compared to other sources, such bone marrow, Wharton jelly, and adipose tissue, MSCs from foreskin harbor several patterns of genes and miRNAs potentially involved in the regulation of inflammatory and immunological responses. These genes are subsequently modulated by inflammation and seem to be involved in regulating the immunological fate of FSK-MSCs [32,33,34,35]. In line with these findings, we have previously shown that an inflammatory milieu has a significant impact on the molecular profile of skin-derived precursors [14] and adult-derived human liver stromal/stem/progenitor cells [63]. These immunological changes need to be further characterized to guarantee a safe cellular product with consistent quality and high therapeutic efficacy [55].

As several interactions may occur between MSCs and Th17 cells within a proinflammatory microenvironment, we evaluated their in vitro interplay and tried to understand the cellular and molecular changes occurring during these cultures. In particular, we analyzed the secretome linked to such coculture, which may be beneficial for inflammation management and tissue repair. MSCs from both bone marrow and adipose tissue were reported to significantly modulate Th17 markers (RORγt, IL-17A, and IL-23R), with some differences compared to FSK-MSCs. The cellular and molecular interactions between MSCs and Th17 cells depend on other parameters, such as inflammation and the cell ratio, regardless of the source of MSCs [22,26]. In vitro cocultures of either primed or non-primed BM-MSCs and AT-MSCs with activated T cells significantly induced IL-17A and RORγt expression, whereas IL-23R expression by T cells was not modulated [64,65]. Herein, only IL-17a expression was induced with FSK-MSCs, whereas no impact was observed on the expression of RORγt and IL-23R. In contrast, all types of MSCs showed substantial changes in the secretome linked to their coculture with T cells. Regardless of their sources, MSCs significantly modulated the Th17 lymphocyte pathway in a complex manner. The secretion profile of 15 cytokines involved in the Th17 immune response (IL-1β, IL-4, IL-6, IL-10, IL-17A, IL-17F, IL-22, IL-21, IL-23, IL-25, IL-31, IL-33, IFN-γ, sCD40, and TNF-α) was significantly influenced by both the cell ratio and inflammation. In a previous study, we reported that an increase in the Th17/Treg ratio was observed when T cells were cocultured with BM-MSCs from multiple myeloma (MM) compared to healthy BM-MSCs [66]. Such shifts in the Th17/Treg balance are likely linked to variations in the secretion profile, with notable increases in IL-6, VCAM-1, and CD40.

More specifically, our data showed that the levels of IL-17A were significantly increased and were the highest in the high (1/5) MSC:T cell ratio coculture containing FSK-MSCs primed with inflammation. However, IL-17A levels showed a slight but significant increase in cocultures containing MSCs primed with inflammation. However, a low (1/80) MSC:T cell ratio showed a modest increase in IL-17A expression regardless of the inflammatory environment. Th17 cells are an IL-17-producing CD4+ T cell subpopulation and are characterized by their ability to release IL-17A, IL-17F, IL-21, and IL-22 [67,68,69]. Recent reports have provided convincing evidence that IL-17-producing T cells play a key role in the pathogenesis of organ-specific autoimmune diseases, a function previously attributed exclusively to IFN-γ-secreting Th1 cells [70]. The differentiation of Th17 cells from naïve T cells looks to implicate the TGF-β, IL-6, IL-21, IL-1b, and IL-23 signaling pathways. Additionally, IL-1α or IL-1β in synergy with IL-23 can stimulate the secretion of IL-17 from memory T cells. The presence of such a proinflammatory environment may greatly influence MSC and T cell biology. The main function of IL-17-secreting T cells is to mediate inflammation by stimulating the production of inflammatory cytokines and chemokines that promote the recruitment of neutrophils and macrophages [70]. Of importance, MSCs need activation or priming signals to participate in tissue repair by facilitating regeneration and immunomodulation [71]. IL-1 primes MSCs toward an anti-inflammatory and protrophic phenotype in vitro [72]. Preactivation of MSCs with TNF-α, IL-1β, and nitric oxide enhances their paracrine effects via a heme oxygenase-1-dependent mechanism, which may help us to maximize the paracrine potential of MSCs [73]. Proinflammatory T cells impair the viability, self-renewal, and differentiation capacities of MSCs [74].

In addition to IL-17, retinoic acid receptor-related orphan nuclear receptor (RORγt) and IL-23R are key players in T cell differentiation into Th17 cells [75]. Accordingly, RORγt influences Th17 differentiation through the regulation of IL-17A, IL-17F, and IL-2 expression [76]. Interestingly, our data showed that RORγt expression levels were significantly decreased regardless of the MSC:T cell ratio with or without inflammation, suggesting that FSK-MSCs may oppose and reduce Th17 differentiation. The latter results seem to be specific to FSK-MSCs since we previously showed that coculture of T cells and stromal/stem cells originating from marrow and adipose tissues constantly increased RORγt expression [64,65]. Accordingly, a decrease in RORγt expression may eventually decrease Th17-related proinflammatory cytokines and thus block the Th17 differentiation pathway. Therefore, knowing that RORγt expression is associated with Th17 polarization and survival, FSK-MSC-mediated inhibition of RORγt expression will negatively influence the shift of Th17 cells to acquire an inflammatory phenotype.

Additionally, IL-23 stimulation of IL-23 receptor (IL-23R)-expressing naïve CD4+ T cell differentiation into inflammatory Th17/ThIL-17 produced a set of inflammatory cytokines, including IL-17, IL-17F, IL-6, and TNF-α, but not IFN-γ and IL-4 [76]. Our results showed that FSK-MSCs at different cell ratios had no detectable influence on IL-23 receptor expression. In addition, the levels of IL-23 cytokines were slightly induced in the coculture of MSCs and T cells compared to the other cytokines, including but not limited to IL-1, IL-6, and IL-17A. Interestingly, comparable results were found in cocultures of T cells and bone marrow- or adipose-derived MSCs, where the levels of both the IL-23 receptor and IL-23 cytokine levels remained unchanged in coculture regardless of the cell ratio and inflammatory state, suggesting the involvement of cytokines other than IL-23 in the proliferation of Th17 cells [65]. Of note, IL-23 signaling might not be required for the induction of Th17 polarization but the expansion of preexisting differentiated Th17-polarized cells. Therefore, induction of RORγt expression by FSK-MSCs maintains and stabilizes the IL-23 expression needed for the expansion of the Th17-polarized pool of cells. While IL-4 and IFN-γ are known to reduce IL-23 receptor expression, the low level of IL-4 in coculture could explain why IL-23 receptor expression remained unchanged. As is known, MSC modulation of the immune response is multifactorial, requiring different signals. Importantly, cytokines exert their immunological effects on cells and tissues in a manner dependent on cytokine identities, combinations, and concentrations. How MSCs modulate these cytokines is also of importance to understand, as it reflects how the immune response is affected. The generation and expansion of Th17 cells depend upon a variety of inflammatory cytokines, including IL-1β, IL-6, IL-21, IL-23, and TGF-β [77]. Importantly, we found that these cytokines were significantly induced in cultures of MSCs primed with inflammation but also remained elevated in cocultures of T cells and MSCs enough to promote Th17 generation from naïve T cells. IL-6 levels are of particular interest since the presence of IL-6 could either favor Th17 generation or be involved in inflammation resolution through a PGE2 induction mechanism [25,78,79]. Furthermore, many inflammatory conditions, e.g., Th17 cell levels in inflamed joints, correlated with high IL-6 levels. Of note, IL-10 levels were elevated in coculture regardless of inflammatory priming, which can participate in keeping the levels of IL-6 below the threshold above which it contributes to inflammation and Th17 generation and expansion.

The tumor necrosis factor transmembrane receptor family (TNFR) member CD40 is transiently upregulated on CD4+ T cell membranes and other cell types in inflammatory and autoimmune illnesses. Additionally, the soluble endogenous form of CD40 (sCD40) acts as a decoy inhibitor of CD154 to CD40, thus modulating both humoral and cellular immunity [80]. Our data with FSK-MSCs corroborated previously obtained results with bone marrow-derived MSCs, where cocultures of a 1:80 T cell:MSC ratio had minimal effect on sCD40 levels significantly induced in T cells activated with inflammation. However, these levels were significantly reduced in cocultures with higher cell ratios [65]. TNF-alpha is a major inflammatory cytokine involved in the etiology of a multitude of inflammatory illnesses [81]. Currently, anti-TNF therapy is a primordial pharmacotherapy involved in the treatment of most autoimmune diseases [81]. Importantly, cocultures of both T cells and MSCs showed reduced levels of TNF in cocultures with a low cell ratio, which was further reduced in cocultures with a high cell ratio, demonstrating an anti-inflammatory effect of FSK-MSCs. However, the mechanisms by which FSK-MSCs inhibit TNF production by stimulated T cells remain to be identified. All these cytokines may differentially regulate Th17 cells and thus drive qualitatively different contributions to the development of inflammation depending on the pathogen and affected tissue. Once generated, Th17 cells have the ability to produce several effector molecules, such as IL-17A, IL-17F, IL-21, IL-22, IL-25, IL-31, and IL-33, that contribute to the progression and pathogenesis of several autoimmune and inflammatory diseases [82]. High proinflammatory cytokine concentrations are thought to promote an MSC2 phenotype, while an MSC1 phenotype may result from low levels of such cytokines [83]. IL-17A and IL-17F cytokines were mainly secreted by activated T cells, with insignificant induction being observed in inflammatory primed MSCs. However, coculture with MSCs substantially increased the levels of IL-17A and IL-17F secretions regardless of the cell ratio or inflammatory priming. The Th17 response was reported to be modulated by adipose tissue (AT)-MSCs in a passage-dependent fashion [84]. AT-MSCs from a late passage (P8) upregulated the Th17 ratio and their abilities to produce IL-17, whereas those from an early passage (P3) had the opposite effect. Darlington et al. observed significant increases in Th17 proliferation and IL-17A secretion induced by MSCs, which were further accentuated when pretreating hMSCs with the proinflammatory cytokine IL-1β [85]. Accordingly, a PGE2-dependent and myeloid cell-mediated mechanism by which MSCs can reciprocally enhance Th17 responses and IL-17 secretion while suppressing Th1 responses was described by Rozenberg et al. Preexposure of MSCs to IL-1β accentuated their capacity to reciprocally regulate Th1 and Th17 responses [86]. These results suggest that MSCs may participate in efficient pathogen elimination by regulating the functional activation of neutrophils through the modulation of IL-17-secreting T cells [87]. Such an increase in cytokines will further promote the formation of an environment suitable for the inflammatory response. These cytokines may thus regulate several different immune and non-immune cells to produce proinflammatory mediators such as chemokines (CXCL1 and CXCL8), which may attract inflammatory cells (neutrophils) and cytokines (IL-6), promoting the activation of Th17 cells. Moreover, Th17 cells stimulate other cells to release the cytokines IL-21 and IL-23, which in turn can induce the differentiation or development of Th17 cells [88]. In addition to their previous roles in the Th17 immune pathway response, IL-21 also regulates both innate and adaptive immune responses, and it not only has key roles in antitumor and antiviral responses but also exerts major effects on inflammatory responses that promote the development of autoimmune diseases and inflammatory disorders. Indeed, it costimulates T and natural killer (NK) cell proliferation and function and regulates B cell survival and differentiation and the function of dendritic cells, suggesting that IL-21 may represent a potentially useful agent for the development of tumor immunotherapies [89]. In our coculture system, the expression and release of several other important pro- and anti-inflammatory cytokines, including IL-10, IL-21, IL-22, IL-23, IL-25, and IFN, were also modulated, but, interestingly, their expression levels mirrored those obtained with coculture with bone marrow-derived mesenchymal stromal/stem cells. While IL-31 production was reduced in cocultures of bone marrow-derived MSCs regardless of the inflammatory environment, the levels of IL-31 remained significantly elevated in cocultures of FSK-MSCs and T-cells, and there was no effect of inflammatory stimulation in this regard. Elevated levels remained elevated in cocultures. Therefore, variations in IL-31 production levels among different types of stromal/stem cells, including FSK-MSCs, will further emphasize the uniqueness of the immunomodulatory properties of FSK-MSCs. The latter data demonstrate that although FSK-MSCs may share some immunomodulatory properties similar to those of other types of MSCs, e.g., bone marrow-derived MSCs, they may have distinct supplementary effects. Overall, the effects of MSCs on the Th17 cell effector pathway appear to be suppressive under specific conditions and sometimes become promoting under other circumstances. In an inflammatory environment, Ghannam et al. reported that increased adhesion of MSCs to Th17 cells induced regulatory characteristics by downregulating the expression of RORγt and upregulating that of FOXP3 [90]. Obermajer and colleagues showed that cells of the Th17 type, predominantly associated with the rejection of allogeneic solid organ grafts, can be directly converted toward a specific subset of T cells, IL-17A+Foxp3+, suggesting a complex mechanism for immunotolerance [91]. MSCs may act as mediators of immune tolerance without complete immunosuppression, which may have significant implications for therapeutic applications. These results suggest that the therapeutic use of MSCs in vivo might exert opposing effects on disease activity, according to the time of therapeutic application and the level of effector T cell activation. Indeed, at day 3 of polarizing conditions, MSCs markedly promoted the expansion of IL-17-secreting Th17 cells while suppressing Th1 differentiation [92].

This study points out for the first time the response and characteristics of FSK-MSCs with regard to the Th17 pathway following their in vitro coculture with lymphocytes. These observations are encouraging and must be extended to further define findings in a more physiological model. As such, the coculture system used herein should be developed and improved. From this perspective, we highlight some factors that we believe are critical in terms of therapeutic success employing cultured FSK-MSCs. The culture medium composition, the cell type and ratio in cocultures, the responder immune population, the source of MSCs, the donor characteristics, and the tissue environment challenges are among the major elements that may affect the outcome of the study. Of importance, it will be of benefit to develop a culture of FSK-MSCs in the presence of human serum alternatives rather than xenogeneic FBS. This step will promote the transition toward the production of FSK-MSCs for clinical use. Such production requires adherence to current good manufacturing practice (cGMP) standards to ensure product quality and safety as well as the standardization and reproducibility of the methods. 

Most biological properties of MSCs are derived from studies with cells expanded ex vivo rather than in in vivo research. For the in vivo investigation of MSCs, different animal models are widely used, but this is not sufficient to properly reflect what is happening in the human body. Given the complexity of MSC–host interactions, it is also imperative to develop screening tools that account for as many variables as possible and precisely predict the in vivo response rates before MSCs enter the body [93]. Selecting the right donor population, choosing the best culture conditions, and picking the patient population that is most likely to give a favorable therapeutic response should also be considered. Thus, the inflammatory environment may modify the fate of MSCs and therefore their therapeutic functions. Feasible approaches are thus needed to monitor the inflammatory status of patients before the infusion of MSCs, and relevant biomarkers indicating the effectiveness of MSCs should be developed, particularly when they are being used in patients with inflammatory and immune diseases [94]. 

## 5. Conclusions

FSK-MSCs may promote immunomodulation and tissue repair through different mechanisms. They act by sensing the tissue environment as well as by modulating the features of local immune and progenitor cells. We observed that complex in vitro cellular and molecular changes occur during these interactions. These changes involve mainly the modulation of Th17 cell markers (IL-17a, IL-23R, RORγt) as well as their cytokine pathway. Of interest, the cell ratio used for the coculture, as well as the presence of an inflammatory setting, significantly influenced the profile of the modulation by FSK-MSCs. The secretome of the coculture holds great importance, as it defines the beneficial effects of MSCs and may become a new and promising alternative for immune-mediated inflammatory diseases and regenerative medicine. A defined secretome signature would support the identification of a target mediator to improve the therapeutic efficiency of FSK-MSCs (by applying an on/off switch strategy) or a relevant biomarker that indicates a specific immunotrophic profile. It is conceivable that, in the near future, details about the in vivo interplay between FSK-MSCs and their environment as well as their physiological interplay during tissue repair will accelerate progress in the field of immunomodulation and tissue remodeling. These observations will strengthen our knowledge about the molecular mechanisms that may improve or hamper the therapeutic potential of FSK-MSCs. These immunological modifications need to be further characterized to guarantee a safe cellular product with consistent quality and high therapeutic efficacy.

## Figures and Tables

**Table 1:** List of monoclonal antibodies for flow cytometry.

Human Primary Antibody	Species	Dilution	Source	Isotype Control
anti-CD73-APC	mouse	1/20	BD Biosciences	APC mouse IgG1
anti-CD90-PE	mouse	1/20	R&D Systems	PE mouse IgG2A
anti-CD105-FITC	mouse	1/20	BioLegend	FITC mouse IgG1
anti-CD34-PC5	mouse	1/20	BD Biosciences	PC5 mouse IgG1
anti-CD14-PE	mouse	1/21	BD Biosciences	PE mouse IgG2a
anti-D19-PE	mouse	1/22	BD Biosciences	PE mouse IgG1
anti-CD45-PC7	mouse	1/20	BD Biosciences	PC7 mouse IgG2a
anti-HLA-DR-PerCP	mouse	1/20	BD Biosciences	PerCP mouse IgG2a

**Table 2:** The % expression of MSC markers as determined by flow cytometry.

Markers	HLA-Dr	CD14	CD19	CD73	CD105	CD90	CD34
FSK-MSCs							
1	0.6	1	0.9	99	95	97	1
2	3	0.5	1	97	85	96	4
3	1	0.8	0.5	98	82	95	2
4	0.5	1.3	1.8	94	90	98	3
5	1.5	3	1	96	89	95	2
6	2	1	2	93	97	99	3
7	0.7	2	3	100	92	96	5
X	1.32857143	1.37142857	1.45714286	96.7142857	90	96.5714286	2.85714286
SEM	0.34483556	0.32419445	0.3250327	0.96890428	2	0.57142857	0.5084323

## Data Availability

The data presented in this study might be available depending on the type of demand and use and are linked to authorities’ authorization. A request must be sent to the corresponding author with the permission of all authors.

## References

[1] Merimi M., Lewalle P., Meuleman N., Agha D., El-Kehdy H., Bouhtit F., Ayoub S., Burny A., Fahmi H., Lagneaux L. (2021). Mesenchymal Stem/stromal cell therapeutic features: The bridge between the bench and the clinic. J. Clin. Med..

[2] Najar M., Bouhtit F., Melki R., Afif H., Hamal A., Fahmi H., Merimi M., Lagneaux L. (2019). Mesenchymal stromal cell-based therapy: New perspectives and challenges. J. Clin. Med..

[3] Rameshwar P. (2009). Microenvironment at tissue injury, a key focus for efficient stem cell therapy: A discussion of mesenchymal stem cells. World J. Stem Cells.

[4] Herzig M.C., Christy B.A., Montgomery R.K., Delavan C.P., Jensen K.J., Lovelace S.E., Cantu C., Salgado C.L., Cap A.P., Bynum J.A. (2021). Interactions of human mesenchymal stromal cells with peripheral blood mononuclear cells in a Mitogenic proliferation assay. J. Immunol. Methods.

[5] Taşlı P., Somuncu O., Turan R.D., Kocabas F., Sahin F. (2015). Immunomodulatory Properties of Human Newborn Foreskin Stem Cells. https://pubmed.ncbi.nlm.nih.gov/20712449/.

[6] Merimi M., Buyl K., Daassi D., Rodrigues R., Melki R., Lewalle P., Vanhaecke T., Fahmi H., Rogiers V., Lagneaux L. (2021). Transcriptional profile of cytokines, regulatory mediators and TLR in mesenchymal stromal cells after inflammatory signaling and cell-passaging. Int. J. Mol. Sci..

[7] Farkhad N.K., Mahmoudi A., Mahdipour E. (2021). How similar are human mesenchymal stem cells derived from different origins? a review of comparative studies. Curr. Stem Cell Res. Ther..

[8] Najar M., Lagneaux L. (2017). Foreskin as a source of immunotherapeutic mesenchymal stromal cells. Immunotherapy.

[9] Najar M., Raicevic G., André T., Fayyad-Kazan H., Pieters K., Bron D., Toungouz M., Lagneaux L. (2016). Mesenchymal stromal cells from the foreskin: Tissue isolation, cell characterization and immunobiological properties. Cytotherapy.

[10] Leung S., Liu X., Fang L., Chen X., Guo T., Zhang J. (2010). The cytokine milieu in the interplay of pathogenic Th1/Th17 cells and regulatory T cells in autoimmune disease. Cell. Mol. Immunol..

[11] Najar M., Crompot E., Van Grunsven L.A., Dollé L., Lagneaux L. (2018). Foreskin-derived mesenchymal stromal cells with aldehyde dehydrogenase activity: Isolation and gene profiling. BMC Cell Biol..

[12] Dominici M., Le Blanc K., Mueller I., Slaper-Cortenbach I., Marini F., Krause D., Deans R., Keating A., Prockop D., Horwitz E. (2006). Minimal criteria for defining multipotent mesenchymal stromal cells. The International Society for Cellular Therapy position statement. Cytotherapy.

[13] Najar M., Raicevic G., Jebbawi F., De Bruyn C., Meuleman N., Bron D., Toungouz M., Lagneaux L. (2012). Characterization and functionality of the CD200–CD200R system during mesenchymal stromal cell interactions with T-lymphocytes. Immunol. Lett..

[14] De Kock J., Rodrigues R.M., Branson S., Verhoye L., Colemonts-Vroninks H., Rombaut M., Boeckmans J., Neuckermans J., Lequeue S., Buyl K. (2020). Inflammation alters the secretome and immunomodulatory properties of human skin-derived precursor cells. Cells.

[15] Kozlowska U., Krawczenko A., Futoma K., Jurek T., Rorat M., Patrzalek D., Klimczak A. (2019). Similarities and differences between mesenchymal stem/progenitor cells derived from various human tissues. World J. Stem Cells.

[16] Pievani A., Scagliotti V., Russo F., Azario I., Rambaldi B., Sacchetti B., Marzorati S., Erba E., Giudici G., Riminucci M. (2014). Comparative analysis of multilineage properties of mesenchymal stromal cells derived from fetal sources shows an advantage of mesenchymal stromal cells isolated from cord blood in chondrogenic differentiation potential. Cytotherapy.

[17] Heo J.S., Choi Y., Kim H.-S., Kim H.O. (2016). Comparison of molecular profiles of human mesenchymal stem cells derived from bone marrow, umbilical cord blood, placenta and adipose tissue. Int. J. Mol. Med..

[18] Najar M., Raicevic G., Kazan H.F., De Bruyn C., Bron D., Toungouz M., Lagneaux L. (2012). Immune-related antigens, surface molecules and regulatory factors in human-derived mesenchymal stromal cells: The expression and impact of inflammatory priming. Stem Cell Rev. Rep..

[19] Schmelzer E., McKeel D.T., Gerlach J.C. (2019). Characterization of human mesenchymal stem cells from different tissues and their membrane encasement for prospective transplantation therapies. BioMed Res. Int..

[20] Wang D., Liu N., Xie Y., Song B., Kong S., Sun X. (2020). Different culture method changing CD105 expression in amniotic fluid MSCs without affecting differentiation ability or immune function. J. Cell. Mol. Med..

[21] Roobrouck V.D., Vanuytsel K., Verfaillie C.M. (2011). Concise review: Culture mediated changes in fate and/or potency of stem cells. Stem Cells.

[22] Haack-Sørensen M., Hansen S.K., Hansen L., Gaster M., Hyttel P., Ekblond A., Kastrup J. (2013). Mesenchymal stromal cell phenotype is not influenced by confluence during culture expansion. Stem Cell Rev. Rep..

[23] Yang Y.-H.K., Ogando C.R., See C.W., Chang T.-Y., Barabino G.A. (2018). Changes in phenotype and differentiation potential of human mesenchymal stem cells aging in vitro. Stem Cell Res. Ther..

[24] Bourin P., Bunnell B.A., Casteilla L., Dominici M., Katz A.J., March K.L., Redl H., Rubin J.P., Yoshimura K., Gimble J.M. (2013). Stromal cells from the adipose tissue-derived stromal vascular fraction and culture expanded adipose tissue-derived stromal/stem cells: A joint statement of the International Federation for Adipose Therapeutics and Science (IFATS) and the International Society for Cellular Therapy (ISCT). Cytotherapy.

[25] Najar M., Rouas R., Raicevic G., Boufker H.I., Lewalle P., Meuleman N., Bron D., Toungouz M., Martiat P., Lagneaux L. (2009). Mesenchymal stromal cells promote or suppress the proliferation of T lymphocytes from cord blood and peripheral blood: The importance of low cell ratio and role of interleukin. Cytotherapy.

[26] Wang Y., Chen X., Cao W., Shi Y. (2014). Plasticity of mesenchymal stem cells in immunomodulation: Pathological and therapeutic implications. Nat. Immunol..

[27] Lee O.J., Luk F., Korevaar S.S., Koch T.G., Baan C., Merino A., Hoogduijn M.J. (2020). The Importance of dosing, timing, and (in)activation of adipose tissue-derived mesenchymal stromal cells on their immunomodulatory effects. Stem Cells Dev..

[28] Mckinnirey F., Herbert B., Vesey G., McCracken S. (2021). Immune modulation via adipose derived Mesenchymal Stem cells is driven by donor sex in vitro. Sci. Rep..

[29] Bianconi E., Casadei R., Frabetti F., Ventura C., Facchin F., Canaider S. (2020). Sex-specific transcriptome differences in human adipose mesenchymal stem cells. Genes.

[30] Skubis A., Gola J., Sikora B., Hybiak J., Paul-Samojedny M., Mazurek U., Łos M.J. (2017). Impact of antibiotics on the proliferation and differentiation of human adipose-derived mesenchymal stem cells. Int. J. Mol. Sci..

[31] Khasawneh R.R., Al Sharie A.H., Rub E.A.-E., Serhan A.O., Obeidat H.N. (2019). Addressing the impact of different fetal bovine serum percentages on mesenchymal stem cells biological performance. Mol. Biol. Rep..

[32] Tonarova P., Lochovska K., Pytlik R., Kalbacova M.H. (2021). The impact of various culture conditions on human mesenchymal stromal cells metabolism. Stem Cells Int..

[33] Hoang V.T., Trinh Q.-M., Phuong D.T.M., Bui H.T.H., Hang L.M., Ngan N.T.H., Anh N.T.T., Nhi P.Y., Nhung T.T.H., Lien H.T. (2021). Standardized xeno- and serum-free culture platform enables large-scale expansion of high-quality mesenchymal stem/stromal cells from perinatal and adult tissue sources. Cytotherapy.

[34] Sanz-Nogués C., O’Brien T. (2021). Current Good manufacturing practice considerations for mesenchymal stromal cells as therapeutic agents. Biomater. Biosyst..

[35] Lechanteur C., Briquet A., Bettonville V., Baudoux E., Beguin Y. (2021). MSC Manufacturing for academic clinical trials: From a clinical-grade to a full GMP-compliant process. Cells.

[36] Godthardt K., Heifer C., Jüngerkes F., Bosio A., Knöbel S. (2019). Efficient GMP compliant expansion of mesenchymal stromal cells (MSCs) from umbilical cord, bone marrow and adipose tissue using a closed cultivation system. Cytotherapy.

[37] Wu X., Ma Z., Wu D. (2020). Derivation of clinical-grade mesenchymal stromal cells from umbilical cord under chemically defined culture condition—Platform for future clinical application. Cytotherapy.

[38] Cugno C., Alkhualifi M., Al-Sulaiti A., Guerrouahen B., Al-Khawaga S., Calderone Z. Foreskin DerivedMesenchymal stromal cell FSKMSC: Setting the ground for the clinical grade production of MSC at Sidra’s GMP facility. Proceedings of the Qatar Foundation Annual Research Conference Proceedings.

[39] Nikolits I., Nebel S., Egger D., Kreß S., Kasper C. (2021). Towards physiologic culture approaches to improve standard cultivation of mesenchymal stem cells. Cells.

[40] Uccelli A., Pistoia V., Moretta L. (2007). Mesenchymal stem cells: A new strategy for immunosuppression?. Trends Immunol..

[41] Tomchuck S.L., Zwezdaryk K.J., Coffelt S.B., Waterman R.S., Danka E.S., Scandurro A.B. (2008). Toll-like receptors on human mesenchymal stem cells drive their migration and immunomodulating responses. Stem Cells.

[42] Reddy P., Ferrara J.L. (2003). Immunobiology of acute graft-versus-host disease. Blood Rev..

[43] Michael S., Achilleos C., Panayiotou T., Strati K. (2016). Inflammation shapes stem cells and stemness during infection and beyond. Front. Cell Dev. Biol..

[44] Crop M.J., Baan C.C., Korevaar S.S., Ijzermans J.N.M., Pescatori M., Stubbs A.P., Van Ijcken W.F.J., Dahlke M.H., Eggenhofer E., Weimar W. (2010). Inflammatory conditions affect gene expression and function of human adipose tissue-derived mesenchymal stem cells. Clin. Exp. Immunol..

[45] Hemeda H., Jakob M., Ludwig A.-K., Giebel B., Lang S., Brandau S. (2010). Interferon-γ and tumor necrosis factor-α differentially affect cytokine expression and migration properties of mesenchymal stem cells. Stem Cells Dev..

[46] Prasanna S.J., Gopalakrishnan D., Shankar S.R., Vasandan A.B. (2010). Pro-inflammatory cytokines, IFNγ and TNFα, influence immune properties of human bone marrow and Wharton Jelly Mesenchymal stem cells differentially. PLoS ONE.

[47] Wright T.M. (1997). Cytokines in acute and chronic inflammation. Front. Biosci..

[48] Daxecker H., Raab M., Markovic S., Karimi A., Griesmacher A., Mueller M.M. (2002). Endothelial adhesion molecule expression in an in vitro model of inflammation. Clin. Chim. Acta.

[49] Hoogduijn M.J., Popp F., Verbeek R., Masoodi M., Nicolaou A., Baan C., Dahlke M.-H. (2010). The immunomodulatory properties of mesenchymal stem cells and their use for immunotherapy. Int. Immunopharmacol..

[50] Mailliard R.B., Wankowicz-Kalinska A., Cai Q., Wesa A., Hilkens C.M., Kapsenberg M.L., Kirkwood J.M., Storkus W.J., Kalinski P. (2004). α-Type-1 polarized dendritic cells: A novel immunization tool with optimized CTL-inducing activity. Cancer Res..

[51] Chen G., Goeddel D.V. (2002). TNF-R1 Signaling: A beautiful pathway. Science.

[52] Last-Barney K., Homon A.C., Faanes R.B., Merluzzi V.J. (1988). Synergistic and overlapping activities of tumor necrosis factor-alpha and IL. J. Immunol..

[53] Schroder K., Hertzog P.J., Ravasi T., Hume D.A. (2004). Interferon-γ: An overview of signals, mechanisms and functions. J. Leukoc. Biol..

[54] Najar M., Raicevic G., Boufker H.I., Stamatopoulos B., De Bruyn C., Meuleman N., Bron D., Toungouz M., Lagneaux L. (2010). Modulated expression of adhesion molecules and galectin-1: Role during mesenchymal stromal cell immunoregulatory functions. Exp. Hematol..

[55] Buyl K., Merimi M., Rodrigues R., Agha D.M., Melki R., Vanhaecke T., Bron D., Lewalle P., Meuleman N., Fahmi H. (2020). The Impact of cell-expansion and inflammation on the immune-biology of human adipose tissue-derived mesenchymal stromal cells. J. Clin. Med..

[56] Raicevic G., Najar M., Stamatopoulos B., De Bruyn C., Meuleman N., Bron D., Toungouz M., Lagneaux L. (2011). The source of human mesenchymal stromal cells influences their TLR profile as well as their functional properties. Cell. Immunol..

[57] El-Kehdy H., Sargiacomo C., Fayyad-Kazan M., Fayyad-Kazan H., Lombard C., Lagneaux L., Sokal E., Najar M., Najimi M. (2017). Immunoprofiling of adult-derived human liver stem/progenitor cells: Impact of hepatogenic differentiation and inflammation. Stem Cells Int..

[58] Raicevic G., Najar M., Pieters K., De Bruyn C., Meuleman N., Bron D., Toungouz M., Lagneaux L. (2012). Inflammation and toll-like receptor ligation differentially affect the osteogenic potential of human mesenchymal stromal cells depending on their tissue origin. Tissue Eng. Part A.

[59] Zhou T., Yuan Z., Weng J., Pei D., Du X., He C., Lai P. (2021). Challenges and advances in clinical applications of mesenchymal stromal cells. J. Hematol. Oncol..

[60] Wang M., Yuan Q., Xie L. (2018). Mesenchymal stem cell-based immunomodulation: Properties and clinical application. Stem Cells Int..

[61] Kim N., Cho S.-G. (2016). Overcoming immunoregulatory plasticity of mesenchymal stem cells for accelerated clinical applications. Int. J. Hematol..

[62] Ma L., Zhang H., Hu K., Lv G., Fu Y., Ayana D.A., Zhao P., Jiang Y. (2015). The imbalance between Tregs, Th17 cells and inflammatory cytokines among renal transplant recipients. BMC Immunol..

[63] El-Kehdy H., Najar M., De Kock J., Agha D.M., Rogiers V., Merimi M., Lagneaux L., Sokal E.M., Najimi M. (2020). Inflammation Differentially modulates the biological features of adult derived human liver stem/progenitor cells. Cells.

[64] Najar M., Fayyad-Kazan H., Faour W.H., Merimi M., Sokal E., Lombard C.A., Fahmi H. (2019). Immunological modulation following bone marrow-derived mesenchymal stromal cells and Th17 lymphocyte co-cultures. Inflamm. Res..

[65] Najar M., Lombard C.A., Fayyad-Kazan H., Faour W.H., Merimi M., Sokal E., Lagneaux L., Fahmi H. (2019). Th17 immune response to adipose tissue-derived mesenchymal stromal cells. J. Cell. Physiol..

[66] André T., Najar M., Stamatopoulos B., Pieters K., Pradier O., Bron D., Meuleman N., Lagneaux L. (2015). Immune impairments in multiple myeloma bone marrow mesenchymal stromal cells. Cancer Immunol. Immunother..

[67] Darlington P.J., Boivin M.-N., Renoux C., François M., Galipeau J., Freedman M.S., Atkins H.L., Cohen J.A., Solchaga L., Bar-Or A. (2010). Reciprocal Th1 and Th17 regulation by mesenchymal stem cells: Implication for multiple sclerosis. Ann. Neurol..

[68] Lai K., Zeng K., Zeng F., Wei J., Tan G. (2011). Allogeneic adipose-derived stem cells suppress Th17 lymphocytes in patients with active lupus in vitro. Acta Biochim. Biophys. Sin..

[69] Murphy C.A., Langrish C.L., Chen Y., Blumenschein W., McClanahan T., Kastelein R.A., Sedgwick J.D., Cua D.J. (2003). Divergent pro- and antiinflammatory roles for IL-23 and IL-12 in joint autoimmune inflammation. J. Exp. Med..

[70] Kamali A.N., Noorbakhsh S.M., Hamedifar H., Jadidi-Niaragh F., Yazdani R., Bautista J.M., Azizi G. (2019). A role for Th1-like Th17 cells in the pathogenesis of inflammatory and autoimmune disorders. Mol. Immunol..

[71] Zachar L., Bačenková D., Rosocha J. (2016). Activation, homing, and role of the mesenchymal stem cells in the inflammatory environment. J. Inflamm. Res..

[72] Redondo-Castro E., Cunningham C., Miller J., Martuscelli L., Aoulad-Ali S., Rothwell N.J., Kielty C.M., Allan S.M., Pinteaux E. (2017). Interleukin-1 primes human mesenchymal stem cells towards an anti-inflammatory and pro-trophic phenotype in vitro. Stem Cell Res. Ther..

[73] Chen H., Min X.-H., Wang Q.-Y., Leung F.W., Shi L., Zhou Y., Yu T., Wang C.-M., An G., Sha W. (2015). re-activation of mesenchymal stem cells with TNF-alpha, IL-1beta and nitric oxide enhances its paracrine effects on radiation-induced intestinal injury. Sci. Rep..

[74] De Cássia Noronha N., Mizukami A., Caliári-Oliveira C., Cominal J.G., Rocha J.L.M., Covas D.T., Swiech K., Malmegrim K.C.R. (2019). Priming approaches to improve the efficacy of mesenchymal stromal cell-based therapies. Stem Cell Res. Ther..

[75] Ivanov I.I., McKenzie B.S., Zhou L., Tadokoro C.E., Lepelley A., Lafaille J.J., Cua D.J., Littman D.R. (2006). The orphan nuclear receptor RORγt directs the differentiation program of proinflammatory IL-17+ T helper cells. Cell.

[76] Iwakura Y. (2006). The IL-23/IL-17 axis in inflammation. J. Clin. Investig..

[77] Martinez G., Nurieva R.I., Yang X.O., Dong C. (2008). Regulation and function of proinflammatory TH17 cells. Ann. N. Y. Acad. Sci..

[78] Bouffi C., Bony C., Courties G., Jorgensen C., Noël D. (2010). IL-6-Dependent PGE2 secretion by mesenchymal stem cells inhibits local inflammation in experimental arthritis. PLoS ONE.

[79] Chen B., Hu J., Liao L., Sun Z., Han Q., Song Z., Zhao R.C. (2010). Flk-1+mesenchymal stem cells aggravate collagen-induced arthritis by up-regulating interleukin. Clin. Exp. Immunol..

[80] Alturaihi H., Hassan G.S., Al-Zoobi L., Salti S., Darif Y., Yacoub D., El Akoum S., Oudghiri M., Merhi Y., Mourad W. (2015). Interaction of CD154 with different receptors and its role in bidirectional signals. Eur. J. Immunol..

[81] Willrich M.A., Murray D.L., Snyder M.R. (2015). Tumor necrosis factor inhibitors: Clinical utility in autoimmune diseases. Transl. Res..

[82] Yasuda K., Takeuchi Y., Hirota K. (2019). The pathogenicity of Th17 cells in autoimmune diseases. Semin. Immunopathol..

[83] Salami F., Tavassoli A., Mehrzad J., Parham A. (2018). Immunomodulatory effects of mesenchymal stem cells on leukocytes with emphasis on neutrophils. Immunobiology.

[84] He X., Zhang Y., Zhu A., Zeng K., Zhang X., Gong L., Peng Y., Lai K., Qu S. (2016). Suppression of interleukin 17 contributes to the immunomodulatory effects of adipose-derived stem cells in a murine model of systemic lupus erythematosus. Immunol. Res..

[85] Laso-García F., Ramos-Cejudo J., Carrillo-Salinas F.J., Ortega L.O., Feliu A., Frutos M.G.-D., Mecha M., Díez-Tejedor E., Guaza C., Gutiérrez-Fernández M. (2018). Therapeutic potential of extracellular vesicles derived from human mesenchymal stem cells in a model of progressive multiple sclerosis. PLoS ONE.

[86] Rozenberg A., Rezk A., Boivin M.-N., Darlington P., Nyirenda M., Li R., Jalili F., Winer R., Artsy E.A., Uccelli A. (2016). Human mesenchymal stem cells impact Th17 and Th1 responses through a prostaglandin E2 and myeloid-dependent mechanism. Stem Cells Transl. Med..

[87] Taghavi-Farahabadi M., Mahmoudi M., Rezaei N., Hashemi S.M. (2020). Wharton’s jelly mesenchymal stem cells exosomes and conditioned media increased neutrophil lifespan and phagocytosis capacity. Immunol. Investig..

[88] Pawankar R., Hayashi M., Yamanishi S., Igarashi T. (2015). The paradigm of cytokine networks in allergic airway inflammation. Curr. Opin. Allergy Clin. Immunol..

[89] Croce M., Rigo V., Ferrini S. (2015). IL-21: A pleiotropic cytokine with potential applications in oncology. J. Immunol. Res..

[90] Ghannam S., Pène J., Torcy-Moquet G., Jorgensen C., Yssel H. (2010). Mesenchymal Stem cells inhibit human Th17 cell differentiation and function and induce a T regulatory cell phenotype. J. Immunol..

[91] Obermajer N., Popp F.C., Soeder Y., Haarer J., Geissler E.K., Schlitt H.J., Dahlke M.H. (2014). Conversion of Th17 into IL-17aneg regulatory T Cells: A novel mechanism in prolonged allograft survival promoted by mesenchymal stem cell–supported minimized immunosuppressive therapy. J. Immunol..

[92] Zhu Z., Han C., Xian S., Zhuang F., Ding F., Zhang W., Liu Y. (2020). Placental mesenchymal stromal cells (PMSCs) and PMSC-derived extracellular vesicles (PMSC-EVs) attenuated renal fibrosis in rats with unilateral ureteral obstruction (UUO) by regulating CD4+ T cell polarization. Stem Cells Int..

[93] Nemeth K. (2014). Mesenchymal stem cell therapy for immune-modulation: The donor, the recipient, and the drugs in-between. Exp. Dermatol..

[94] Wang L.-T., Ting C.-H., Yen M.-L., Liu K.-J., Sytwu H.-K., Wu K.K., Yen B.L. (2016). Human mesenchymal stem cells (MSCs) for treatment towards immune- and inflammation-mediated diseases: Review of current clinical trials. J. Biomed. Sci..

